# Supplementary material for: Red Light Resets the Expression Pattern, Phase, and Period of the Circadian Clock in Plants: A Computational Approach
Source: Biology (Basel). 2022 Oct 9;11(10):1479. doi: 10.3390/biology11101479 (PMC9598827; doi:10.3390/biology11101479)
Supplement: Supplementary file 1 [file biology-11-01479-s001.zip › biology-1927634-supplematary material.pdf]

Red light resets the expression pattern, phase, and period of the circadian clock in plants: a computational approach

Supplementary Material

Ting Huang [1], Yao Shui [2], Yue Wu[2], Xilin Hou[1,*] and Xiong You [2,*]

Page

S1. Differential equations for the red-light-entrained plant circadian clock 3

S2. Model parameterization and sensitivity analysis 4

S2.1 Model parameterization 4

S2.2 Sensitivity analysis of parameters..4

S3. Phase response curve and stability analysis..5

S3.1 Phase response curve 5

S3.2 Steady states and stability analysis 5

S4. Supplementary Figures and Tables 7

Figure S1. The time evolution of CCA1 mRNA levels under skeleton period in WT and *elf3* mutant. 8

Figure S2. *CCA1*/*LHY* and *PRR5*/*TOC1* expression profiles and phase shift in different photoperiods. 9

Figure S3. Simulated expression profiles under the subjectively natural condition. 10

Figure S4. Bifurcation analysis of steady state and sustained oscillation in *CL* and *P51* expression as well as the periods under oscillation areas. 11

Figure S5. Sensitivity analysis for the phase and period of CCA1 mRNA under constant red light. 12

Figure S6. Validation of expression patterns of clock genes under constant red light. 12

Table S1. Core variables for the red-light entrainment plant circadian clock model. 12

Table S2. Basal parameter values used for numerical simulation. 13

Table S3. The predicted periods and phases of clock gen oscillation system induced by diverse red light/dark cycles. 14

S5. Methods of data extraction and numerical simulation 16

S5.1 Data extraction 16

S5.2 Numerical method 16

S6. Computer codes used for numerical simulation 17

S1. Differential equations for the red-light-entrained plant circadian clock

Based on the available compact model of De Caluwé et al. [10], we developed an ordinary differential equation model for the red-light-entrained plant circadian clock as presented in Figure 1. The model contains 11 variables and 55 parameters. Each variables and parameters can be acquired in Section 3 below.

The time evolution of the mRNA and protein levels of the variables CL (CCA1/LHY), P97 (PRR9/PRR7), P51 (PRR5/TOC1), EL (ELF4/LUX), ELF3 and of the activity of P are governed by the following differential equations:

$$\frac{\mathrm{d}[CL]_m}{\mathrm{d}t} = \left(v_1 + v_{1L}\cdot L(t)\cdot [P] + R(t)\cdot\left(v_{1A}\cdot\frac{[EL]_p^2}{K_{3B}^2+[EL]_p^2} + v_{1B}\cdot\frac{[ELF3]_p^2}{K_{3C}^2+[ELF3]_p^2}\right)\right)\cdot \frac{1}{1+\left(\frac{[CL]_p}{K_0}\right)^2+\left(\frac{[P97]_p}{K_1}\right)^2+\left(\frac{[P51]_p}{K_2}\right)^2} - \left(k_{1L}\cdot L(t) + k_{1D}\cdot D(t) + k_{1R}\cdot R(t)\right)\cdot [CL]_m \quad \text{(S1)}$$

$$\frac{\mathrm{d}[CL]_p}{\mathrm{d}t} = \left(p_1 + p_{1L}\cdot L(t)\right)\cdot [CL]_m - \left(d_{1R}\cdot R(t) + d_1\cdot\left(1-R(t)\right)\right)\cdot [CL]_p \quad \text{(S2)}$$

$$\frac{\mathrm{d}[P97]_m}{\mathrm{d}t} = \left(v_2 + v_{2L}\cdot L(t)\cdot [P]\right)\frac{1}{1+\left(\frac{[P51]_p}{K_4}\right)^2+\left(\frac{[EL]_p}{K_5}\right)^2+\left(\frac{[CL]_p}{K_{5b}}\right)^2} - k_2\cdot [P97]_m \quad \text{(S3)}$$

$$\frac{\mathrm{d}[P97]_p}{\mathrm{d}t} = p_2\cdot [P97]_m - \left(d_{2D}\cdot D(t) + d_{2L}\cdot L(t) + d_{2R}\cdot R(t)\right)\cdot [P97]_p \quad \text{(S4)}$$

$$\frac{\mathrm{d}[P51]_m}{\mathrm{d}t} = v_3\cdot\frac{1}{1+\left(\frac{[P51]_p}{K_7}\right)^2+\left(1-R(t)\right)\left(\frac{[CL]_p}{K_6}\right)^2+R(t)\left(\frac{[CL]_p}{K_{6R}}\right)^2} - k_3\cdot [P51]_m \quad \text{(S5)}$$

$$\frac{\mathrm{d}[P51]_p}{\mathrm{d}t} = p_3\cdot [P51]_m - \left(d_{3D}\cdot D(t) + d_{3L}\cdot L(t) + d_{3R}\cdot R(t)\right)\cdot [P51]_p \quad \text{(S6)}$$

$$\frac{\mathrm{d}[EL]_m}{\mathrm{d}t} = \left(v_{4L}\cdot L(t) + v_{4D}\cdot D(t) + v_{4R}\cdot R(t)\right)\cdot\frac{1}{1+\left(\frac{[CL]_p}{K_8}\right)^2+\left(\frac{[P51]_p}{K_9}\right)^2+\left(\frac{[EL]_p}{K_{10}}\right)^2} - k_4\cdot [EL]_m \quad \text{(S7)}$$

$$\frac{\mathrm{d}[EL]_p}{\mathrm{d}t} = p_4\cdot [EL]_m - \left(d_{4D}\cdot D(t) + d_{4L}\cdot L(t) + d_{4R}\cdot R(t)\right)\cdot [EL]_p \quad \text{(S8)}$$

$$\frac{\mathrm{d}[ELF3]_m}{\mathrm{d}t} = \left(v_{5L} \cdot L(t) + v_{5D} \cdot D(t) + v_{5R} \cdot R(t)\right) \cdot \frac{1}{1 + \left(\frac{[CL]_p}{K_{12}}\right)^2} - k_5 \cdot [ELF3]_m \tag{S9}$$

$$\frac{\mathrm{d}[ELF3]_p}{\mathrm{d}t} = p_5 \cdot [ELF3]_m - d_{5R} \cdot R(t) \cdot [ELF3]_p \tag{S10}$$

$$\frac{\mathrm{d}[P]}{\mathrm{d}t} = (v_6 - p_6 \cdot [P]) \cdot D(t) - d_6 \cdot [P] \cdot L(t) \tag{S11}$$

The parameters L, D and R represent white light, darkness, and red light, respectively. Their values are $L = 1$, $D = 0$ and $R = 0$ during white light phases, $L = 0$, $D = 1$ and $R = 0$ during dark phases, and $L = 0$, $D = 0$ and $R = 1$ during red light phases.

S2. Model parameterization and sensitivity analysis

S2.1 Model parameterization

Model development is followed by model calibration, in which experimental data are often capitalized to estimate the kinetic parameters. Of course, some of these parameters can be directed adopted from a published paper. Others can be determined using some databases that store comprehensive information on biochemical reactions and their kinetic properties.

Experimental data are generally the times course of gene expression, containing the features of clock components, such as steady state, amplitude, phase etc. This motivated us to construct a cost function to quantitatively asses the goodness of fit of our solution to qualitative features present in the experiment data. The cost function is defined by

$$\delta = \sum_{i=C,T} (y_i - \overline{y_i})^2 + \sum_{i=C,T} \left(\varphi_i - \varphi_{i_0}\right)^2 + \sum_{i=C,T} \left(\phi_i - \phi_{i_0}\right)^2 + \sum_{i=C,T} \left(PI_i - PI_{i_0}\right)^2$$

$$\coloneqq \delta_{SSE} + \delta_{\varphi} + \delta_{\phi} + \delta_{PI}.$$

where C and T denote the genes *CCA1* and *TOC1*. The cost function is a sum of four terms. Each term is described in turn as follows: δ_{SSE} measures the difference between the model simulated expression and the experimental expression; δ_{φ} and δ_{ϕ} measure the differences between the respect simulated peaks and trough times (phase) and experimentally observed data; δ_{PI} is the difference between the experimental period of *CCA1* and *TOC1* mRNA levels and the period of oscillation predicted by the model.

S2.2 Sensitivity analysis of parameters

Local sensitivity analysis (LSA) and global sensitivity analysis (GSA) are two major approaches to analyzing sensitivity of parameters in a system [67]. Among widely used techniques are differential sensitivity analysis, one at a time sensitivity measures, factorial analysis, correlation analysis, regression analysis and subjective sensitivity analysis in different sensitivity methods. The most fundamental method with differentiation is one at a time sensitivity measure, in which varying parameter values are taken one at a time [68]. One-at-a-time sensitivity measure is also known as a local analysis method since

it aims to gain the addressed point estimation and not the entire distribution. The following steps were followed:
First step: Select a particular parameter to test and define its basal value. All the other parameters of the model remain unchanged.
Second step: Alter the value of the tested parameter until the behavior of the output variable qualitatively changes (e.g., stable steady state changes to sustained oscillation).
Third step: Find the percentage of change in the output of parameters and the percentage of change in the basal value.
Fourth step: Calculate the sensitivity interval of the parameter by dividing the percentage of change in output by the percentage of change in the parameter value with respect to the basal value.
The result of sensitivity analysis of red-light-associated parameter was displayed in Figure S5.

S3. Phase response curve and stability analysis

S3.1 Phase response curve

Phase response curve (PRC) is used to detect the effect of red-light cues on the circadian rhythm. We impose the red−light pulses at different times of the day, and calculated the phase shifts induced by red−light pulses. In order to obtain a stable phase, the model was entrained to L:D=12 h:12 h for 200 h before exposed to red-light stimulus. The difference between the peak time of the pre-disturbed oscillation t_{before} and the peak time of the undisturbed oscillation t_{after} is given by Δt. Then the phase shift $\Delta\phi$ is defined by:

$$\Delta\phi = \frac{\Delta t}{FRP} = \frac{t_{before} - t_{after}}{FRP}$$

where FRP indicates the free-running period.

S3.2 Steady states and stability analysis

The existence and stability of the steady states of a system determined the dynamical behavior of that system. We calculated the steady state of eq. (S1) – (S10) in constant red-light free-running treatment, and analyzed its stability by Jacobi matrix whose entries are given below.

$$\frac{\partial f_1}{\partial [CL]_m} = -k_{1R},$$

$$\frac{\partial f_1}{\partial [CL]_p} = \frac{2[CL]_p\left(v_1+\left(v_{1A}\cdot\frac{[EL]_p^2}{K_{3B}^2+[EL]_p^2}+v_{1B}\cdot\frac{[ELF3]_p^2}{K_{3C}^2+[ELF3]_p^2}\right)\right)}{{K_0}^2\left(1+\left(\frac{[CL]_p}{K_0}\right)^2+\left(\frac{[P97]_p}{K_1}\right)^2+\left(\frac{[P51]_p}{K_2}\right)^2\right)^2},$$

$$\frac{\partial f_1}{\partial [P97]_p} = \frac{2[P97]_p\left(v_1+\left(v_{1A}\cdot\frac{[EL]_p^2}{K_{3B}^2+[EL]_p^2}+v_{1B}\cdot\frac{[ELF3]_p^2}{K_{3C}^2+[ELF3]_p^2}\right)\right)}{{K_1}^2\left(1+\left(\frac{[CL]_p}{K_0}\right)^2+\left(\frac{[P97]_p}{K_1}\right)^2+\left(\frac{[P51]_p}{K_2}\right)^2\right)^2},$$

$$\frac{\partial f_1}{\partial [P51]_p} = \frac{2[P51]_p\left(v_1+\left(v_{1A}\cdot\frac{[EL]_p^2}{K_{3B}^2+[EL]_p^2}+v_{1B}\cdot\frac{[ELF3]_p^2}{K_{3C}^2+[ELF3]_p^2}\right)\right)}{{K_2}^2\left(1+\left(\frac{[CL]_p}{K_0}\right)^2+\left(\frac{[P97]_p}{K_1}\right)^2+\left(\frac{[P51]_p}{K_2}\right)^2\right)^2},$$

$$\frac{\partial f_1}{\partial [EL]_p} = \frac{v_{1A}\cdot\frac{2[EL]_p}{\left(K_{3B}^2+[EL]_p^2\right)^2}}{1+\left(\frac{[CL]_p}{K_0}\right)^2+\left(\frac{[P97]_p}{K_1}\right)^2+\left(\frac{[P51]_p}{K_2}\right)^2},$$

$$\frac{\partial f_1}{\partial [ELF3]_p} = \frac{v_{1B}\cdot\frac{2[ELF3]_p}{\left(K_{3C}^2+[ELF3]_p^2\right)^2}}{1+\left(\frac{[CL]_p}{K_0}\right)^2+\left(\frac{[P97]_p}{K_1}\right)^2+\left(\frac{[P51]_p}{K_2}\right)^2},$$

$$\frac{\partial f_2}{\partial [CL]_m} = p_1,$$

$$\frac{\partial f_2}{\partial [CL]_p} = -d_{1R},$$

$$\frac{\partial f_3}{\partial [CL]_p} = \frac{2v_2[CL]_p}{{K_{5b}}^2\left(1+\left(\frac{[P51]_p}{K_4}\right)^2+\left(\frac{[EL]_p}{K_5}\right)^2+\left(\frac{[CL]_p}{K_{5b}}\right)^2\right)^2},$$

$$\frac{\partial f_3}{\partial [P97]_m} = -k_2,$$

$$\frac{\partial f_3}{\partial [P51]_p} = \frac{2v_2[P51]_p}{{K_4}^2\left(1+\left(\frac{[P51]_p}{K_4}\right)^2+\left(\frac{[EL]_p}{K_5}\right)^2+\left(\frac{[CL]_p}{K_{5b}}\right)^2\right)^2},$$

$$\frac{\partial f_3}{\partial [EL]_p} = \frac{2v_2[EL]_p}{{K_5}^2\left(1+\left(\frac{[P51]_p}{K_4}\right)^2+\left(\frac{[EL]_p}{K_5}\right)^2+\left(\frac{[CL]_p}{K_{5b}}\right)^2\right)^2},$$

$$\frac{\partial f_4}{\partial [P97]_m} = p_2,$$

$$\frac{\partial f_4}{\partial [P97]_p} = -d_{2R},$$

$$\frac{\partial f_5}{\partial [CL]_p} = \frac{2v_3[CL]_p}{{K_{6R}}^2\left(1+\left(\frac{[P51]_p}{K_7}\right)^2+\left(\frac{[CL]_p}{K_{6R}}\right)^2\right)^2},$$

$$\frac{\partial f_5}{\partial [P51]_m} = -k_3,$$

$$\frac{\partial f_5}{\partial [P51]_p} = \frac{2v_3[P51]_p}{{K_7}^2\left(1+\left(\frac{[P51]_p}{K_7}\right)^2+\left(\frac{[CL]_p}{K_{6R}}\right)^2\right)^2},$$

$$\frac{\partial f_6}{\partial [P51]_m} = p_3,$$

$$\frac{\partial f_6}{\partial [P51]_p} = -d_{3R},$$

$$\frac{\partial f_7}{\partial [CL]_p} = \frac{2v_{4R}[CL]_p}{K_8{}^2\left(1+\left(\frac{[CL]_p}{K_8}\right)^2+\left(\frac{[P51]_p}{K_9}\right)^2+\left(\frac{[EL]_p}{K_{10}}\right)^2\right)^2},$$

$$\frac{\partial f_7}{\partial [P51]_p} = \frac{2v_{4R}[P51]_p}{K_9{}^2\left(1+\left(\frac{[CL]_p}{K_8}\right)^2+\left(\frac{[P51]_p}{K_9}\right)^2+\left(\frac{[EL]_p}{K_{10}}\right)^2\right)^2},$$

$$\frac{\partial f_7}{\partial [EL]_m} = -k_4,$$

$$\frac{\partial f_7}{\partial [EL]_p} = \frac{2v_{4R}[EL]_p}{K_{10}{}^2\left(1+\left(\frac{[CL]_p}{K_8}\right)^2+\left(\frac{[P51]_p}{K_9}\right)^2+\left(\frac{[EL]_p}{K_{10}}\right)^2\right)^2},$$

$$\frac{\partial f_8}{\partial [EL]_m} = p_4,$$

$$\frac{\partial f_8}{\partial [EL]_p} = -d_{4R},$$

$$\frac{\partial f_9}{\partial [CL]_p} = \frac{-2v_{5R}[CL]_p}{K_{12}{}^2\left(1+\left(\frac{[CL]_p}{K_{12}}\right)^2\right)^2},$$

$$\frac{\partial f_9}{\partial [ELF3]_m} = -k_5,$$

$$\frac{\partial f_{10}}{\partial [ELF3]_m} = p_5,$$

$$\frac{\partial f_{10}}{\partial [ELF3]_p} = -d_{5R},$$

where the right sides of eq. (S1) – (S10) are enumerated as f_1, f_2, f_3, f_4, f_5, f_6, f_7, f_8, f_9, f_{10}. The steady state is given by

$$(0.81625, 2.0733, 0.47683, 0.42619, 0.57289, 0.24443, 0.30991, 1.11792, 0.68470, 6.0924).$$

The eigenvalues of the Jacobian at the steady state are

-9.2199, 0.5706, $-0.0719 + 0.9148j$, $-0.0719 - 0.9148j$, $-1.2462 + 0.9279j$, $-1.2462 - 0.9279j$, -1.9193, -1.4943, -0.5506, -0.9800.

Since there are one eigenvalue having positive real part and two conjugate complex roots, the model assumes sustained oscillation under constant red light.

S4. Supplementary Figures and Tables

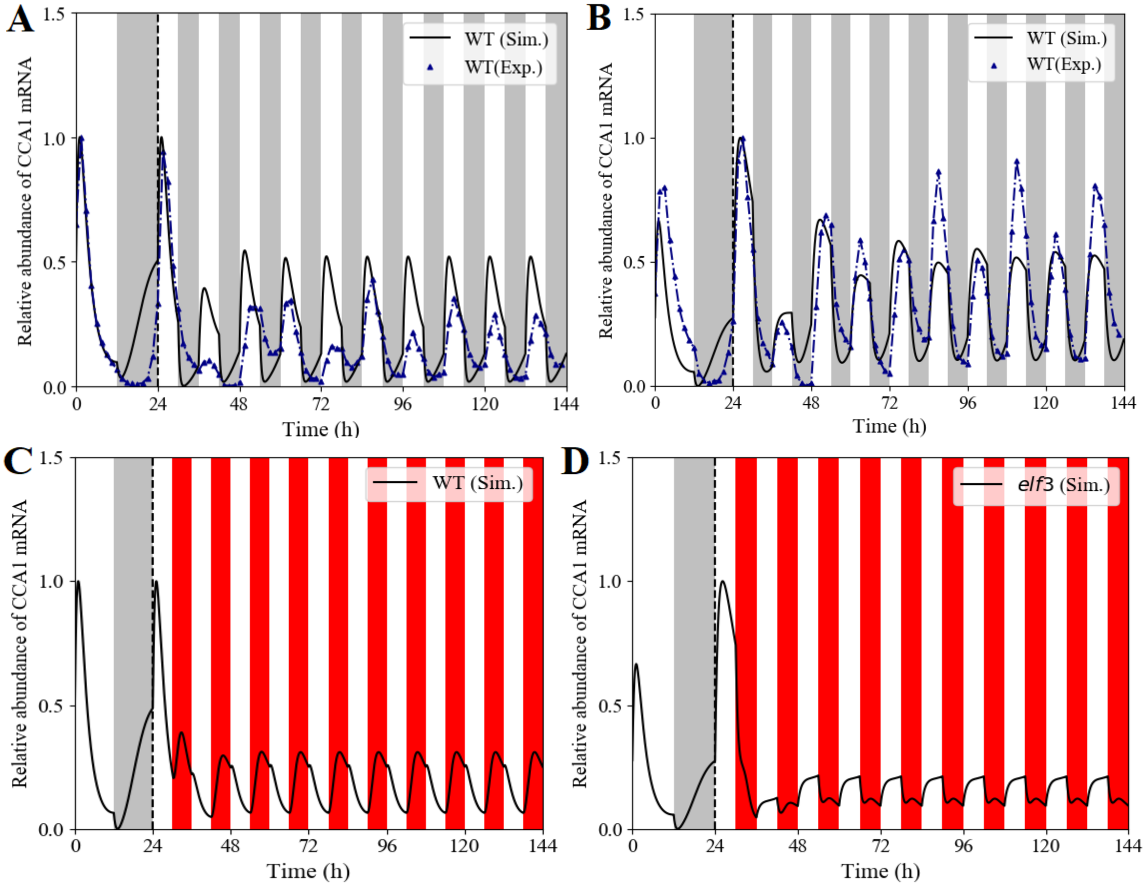

Figure S1. The time evolution of CCA1 mRNA levels under the skeleton period in WT and *elf3* mutant. CCA1 transcriptional level was simulated for 1 day in a 12 h white light/12 h dark cycle, then WT lines were shifted to 6 h light/6 h dark cycles, in either white light (A, B) or red light (C, D). The grey and red bands represent darkness and red-light treatment, respectively. The black solid lines and the blue triangles indicate simulated expression and experimental data, respectively.

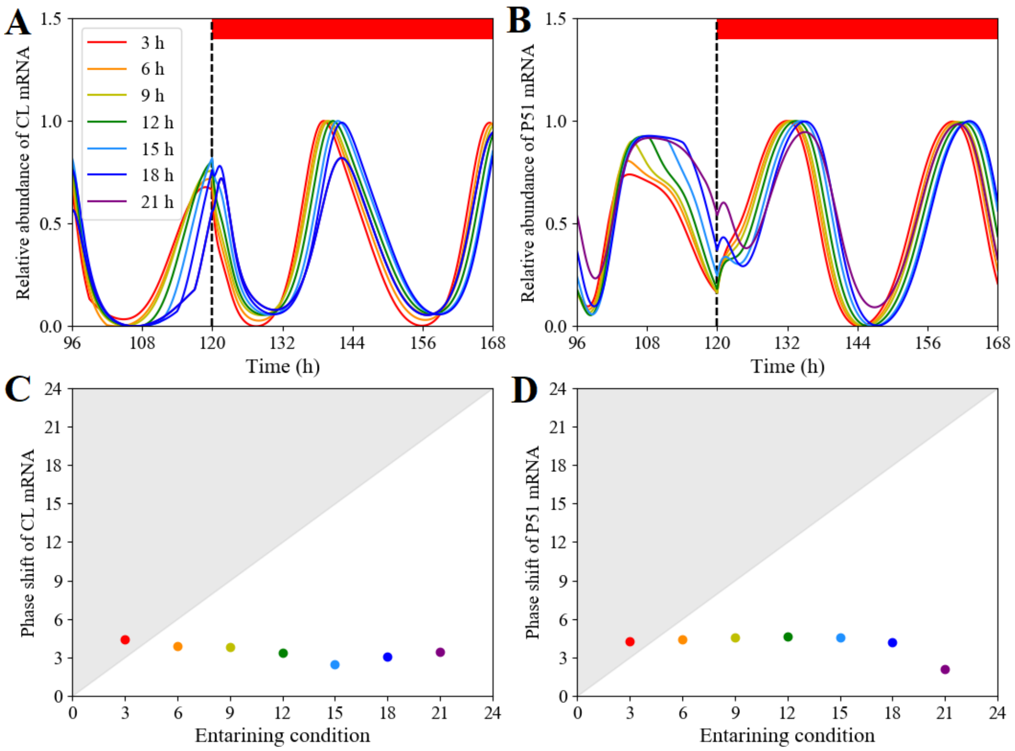

Figure S2. *CCA1*/*LHY* and *PRR5*/*TOC1* expression profiles and phase shifts in different photoperiods. The time courses of *CCA1*/*LHY* (A) and *PRR5*/*TOC1* (B) transcription levels were simulated in photoperiods ranging from 3 to 21 h in increment of 3 h, followed by continuous red-light exposure. The colors changed from red to purple. The phase shifts of *CCA1*/*LHY* (C) and *PRR5*/*TOC1* (D) under different light entraining conditions.

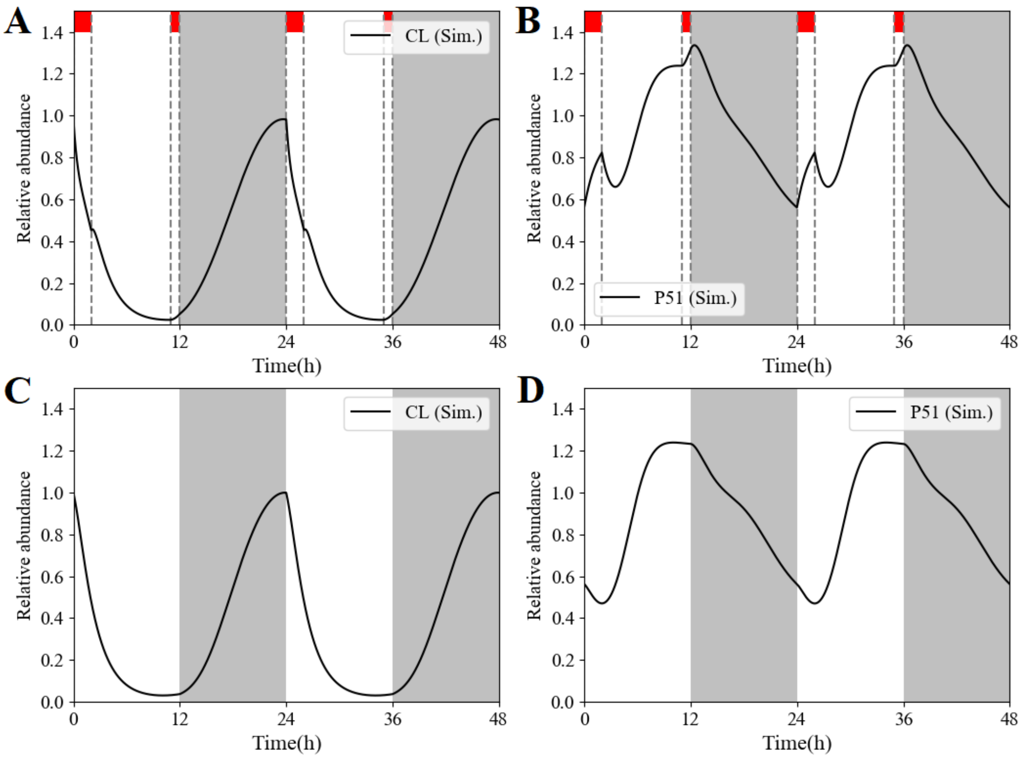

Figure S3. Simulated expression profiles under the subjectively natural condition. (A) and (B) are time evolutions of respective CL mRNA and P51mRNA under the subjectively natural photoperiod. (C) and (D) are time evolutions of respective CL mRNA and P51 mRNA under white light/dark cycles. The grey and red bands represent darkness and red-light treatment, respectively.

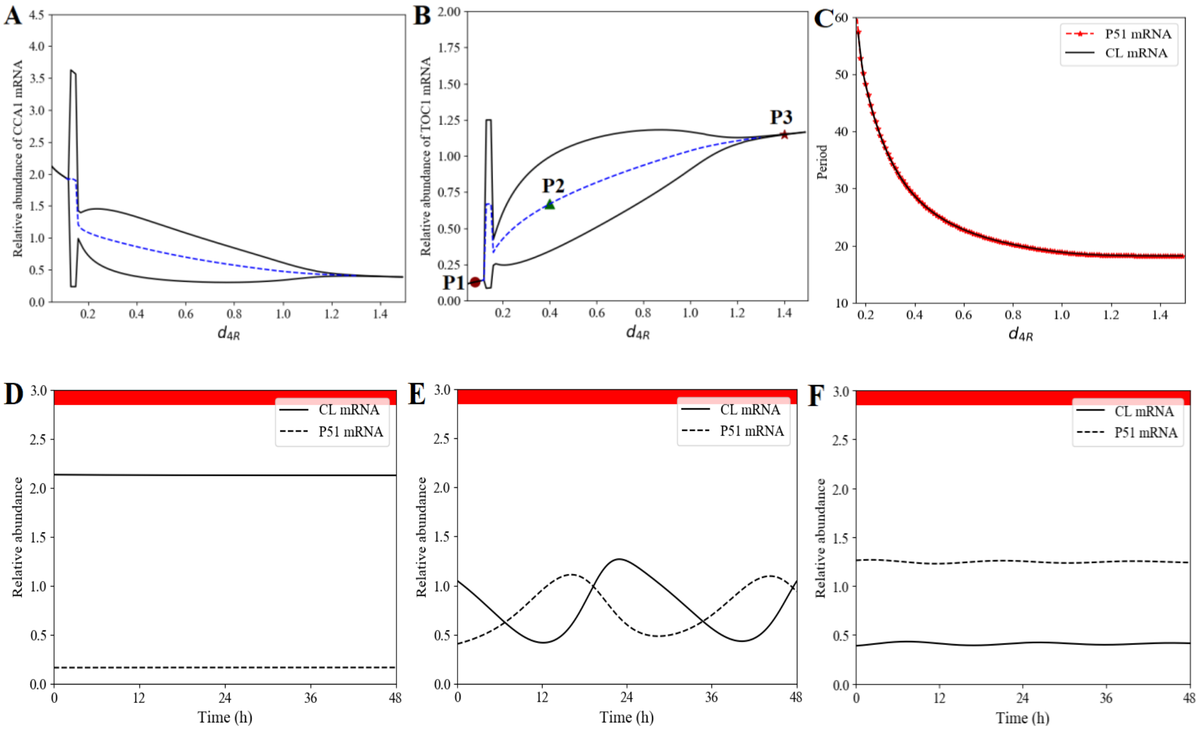

Figure S4. Bifurcation analysis for the model. (A) Bifurcation diagram for *CL* expression with respect to the EL protein degradation rate $\boldsymbol{d_{4R}}$. (A) Bifurcation diagram for *P51* expression with respect to the EL protein degradation rate $\boldsymbol{d_{4R}}$. The blue dotted line represents the unstable steady state. The *CL* (*P51*) mRNA level oscillates in an umbrella region. (C) The dependence of the periods of *CL* and *P51* mRNA oscillations. (D)-(F) Time evolutions of *CL* and *P51* mRNA for $\boldsymbol{d_{4R} = 0.05}$, $\boldsymbol{0.4}$ and$\boldsymbol{1.4}$, corresponding to a high *CL* mRNA and low *P51* mRNA steady state, sustained oscillation in both *CL* mRNA and *P51* mRNA, a low *CL* mRNA and high *P51* mRNA steady state, respectively.

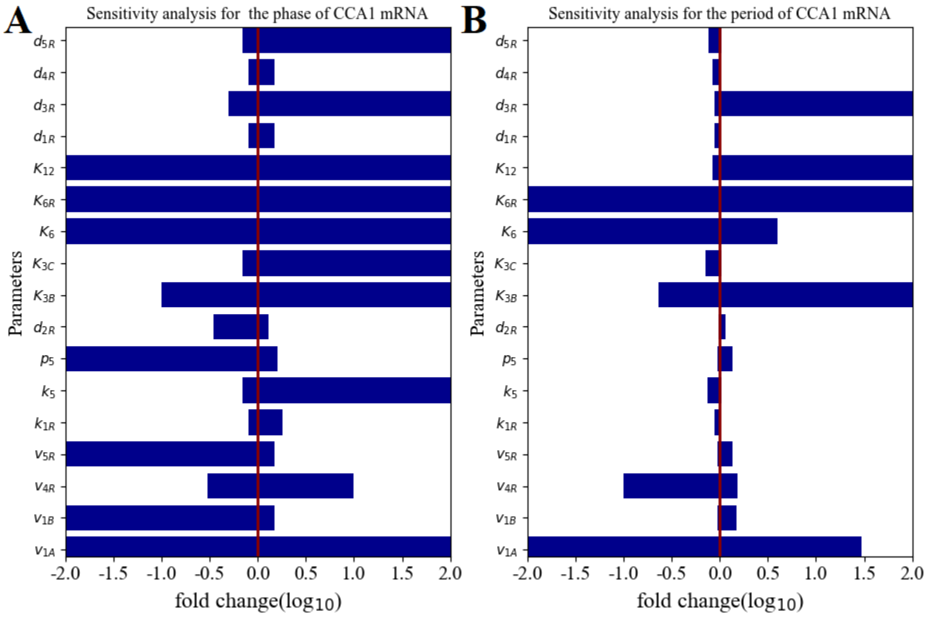

Figure S5. Sensitivity analysis for the phase and period of CCA1 mRNA with respect to red-light-associated parameters under constant red light. In order to assess the sensitivity of the red-light-entrained model to changes in parameter values, we determined for each parameter, the specific range of CCA1 mRNA expression peak time and the period occurrence exposure to red-light free-running, with the basal values listed in Table S2. In the sensitivity analysis, every parameter ranged from $\mathbf{10^{-2}}$ to $\mathbf{10^{2}}$ multiples of the basal values. 17 red-light-associated parameters are listed on the vertical axis. The horizontal axis indicates the logarithmic scale of the fold change in parameter values from −2 to 2. (A) The range of each parameter was determined by the requirement that there were 4 or 5 peaks of *CCA1* mRNA during 120 h to 240 h. (B) The range of each parameter was determined by the requirement that the period of *CCA1* mRNA lay between 26 h and 30 h after constant red-light entrainment.

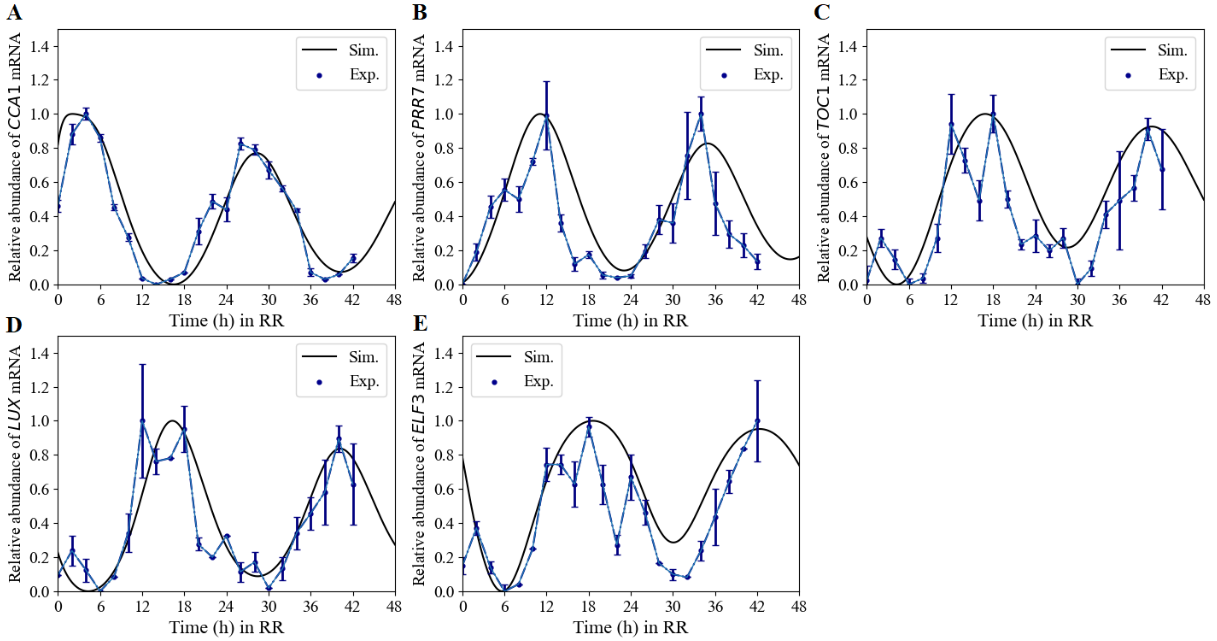

Figure S6. Validation of expression patterns of clock genes under constant red light. The expression profiles were extracted from MacGregor et al. [69]. Plants were grown for 7 d under 12L:12D white light/dark cycles at 22°C followed by 2 d under 12R:12D red light/dark cycles before being released to constant red light (RR) for sampling in 3 biological replicates. Transcript abundance levels were normalized to their maximum value. Experimental data (dots, Exp.) presented are the average ± SE. The simulation results were consistent with the experimental data.

Table S1. Core variables for the red-light entrainment plant circadian clock model.

The first column lists the sequence of variables and the definition of each variable is given in the second column.

Symbol	Definition
$[\boldsymbol{CL}]_{\boldsymbol{m}}$	the concentration of CCA1/LHY mRNA

$[CL]_p$	the concentration of CCA1/LHY protein
$[P97]_m$	the concentration of PRR9/PRR7 mRNA
$[P97]_p$	the concentration of PRR9/PRR7 protein
$[P51]_m$	the concentration of PRR5/TOC1 mRNA
$[P51]_p$	the concentration of PRR5/TOC1 protein
$[EL]_m$	the concentration of ELF4/LUX mRNA
$[EL]_p$	the concentration of ELF4/LUX protein
$[ELF3]_m$	the concentration of ELF3 mRNA
$[ELF3]_p$	the concentration of ELF3 protein
P	The activation of light–sensitive proteins

Table S2. Basal parameter values used for numerical simulation.

The first column lists all the parameters involved in eq. (S1) – (S11). The definition of each parameter is given in the second column, followed by the corresponding values in the third column. The last column indicates the units of parameters.

Parameter	Description	Value	Unit
v_{1A}	EL-induced synthesis of *CCA1/LHY* mRNA	7.22	nM h^{-1}
v_{1B}	ELF3-induced synthesis of *CCA1/LHY* mRNA	8.32	nM h^{-1}
v_{4R}	Red-light-induced synthesis of *ELF4/LUX* mRNA	6.1	nM h^{-1}
v_{5R}	red-light-induced synthesis of *ELF3* mRNA	6.55	nM h^{-1}
v_{5L}	white-light-induced synthesis of *ELF3* mRNA	1.3	nM h^{-1}
v_{5D}	dark-induced synthesis of *ELF3* mRNA	1.3	nM h^{-1}

$\boldsymbol{v_6}$	dark-induced synthesis of *P* protein	0.3	nM h^{-1}
$\boldsymbol{k_{1R}}$	*CCA1/LHY* mRNA degradation (red light)	1.2	h^{-1}
$\boldsymbol{k_5}$	*ELF3* mRNA degradation (red light)	9.22	h^{-1}
$\boldsymbol{p_5}$	ELF3 translation	8.72	h^{-1}
$\boldsymbol{p_6}$	*P* protein degradation (dark)	0.3	h^{-1}
$\boldsymbol{K_{3B}}$	activation of *CCA1/LHY* by ELF4/LUX	5.14	nM
$\boldsymbol{K_{3C}}$	activation of *CCA1/LHY* by ELF3	7.65	nM
$\boldsymbol{K_6}$	inhibition of *PRR5/TOC1* by CCA1/LHY	0.7(white)<br>1.43(red)	nM
$\boldsymbol{K_{6R}}$	inhibition of *PRR5/TOC1* by CCA1/LHY (red light)	1.73	nM
$\boldsymbol{K_{12}}$	activation of *ELF3* by CCA1/LHY	10.7	nM
$\boldsymbol{d_{1R}}$	CCA1/LHY degradation (red light)	0.44	h^{-1}
$\boldsymbol{d_{2R}}$	PRR9/ PRR7 degradation (red light)	1.13	h^{-1}
$\boldsymbol{d_{3R}}$	PRR5/TOC1 degradation (red light)	1.5	h^{-1}
$\boldsymbol{d_{4R}}$	ELF4/LUX degradation (red light)	0.42	h^{-1}
$\boldsymbol{d_{5R}}$	ELF3 degradation (red light)	0.98	h^{-1}
$\boldsymbol{d_6}$	*P* protein degradation (white light)	1	h^{-1}

Table S3. The predicted periods and phases of clock gene oscillation induced by diverse red light/dark cycles.

Light cycles	Phase (h) of *CL* mRNA	Phase (h) of *P51* mRNA	Period (h)
**constant red light**	2.5	18.27	26.4
**8 h red light/16 h dark**	1.67	16.48	24
**12 h red light/12 h dark**	2.32	1.54	24
**16-h red-light/8 h dark**	17.67	6	24.6
**3 h red light/21 h dark**	23.9	9.75	24
**6 h red light/18 h dark**	21.71	10.07	24.02
**9 h red light/15 h dark**	21.78	11.661	24.03
**15 h red light/9 h dark**	12.5	1.11	24.5
**18 h red light/6 h dark**	15.62	4.81	24.66
**21 h red light/3 h dark**	15.27	8.71	22.33
**3 h red light/9 h white light/12 h dark**	21.74	8.12	24
**6 h red light/6 h white light/12 h dark**	21.82	8.86	24
**9 h red light/3 h white light/12 h dark**	22.12	12.33	24
**3 h white light/9 h red light/12 h dark**	10.33	4.29	24
**6 h white light/6 h red light/12 h dark**	11.01	5.44	24
**9 h white light/3 h red light/12 h dark**	14.72	7.71	24

S5. Methods of data extraction and numerical simulation

S5.1 Data extraction

In order to prove the validity of the core model, it is necessary to compare the numerical simulation results with the experimental data. We extracted the experimental data by means of the software *ImageJ* for Image Processing and Analysis (https://imagej.nih.gov/ij/). The following steps were followed:

First step: Download the required experimental data graphs in the form of bar chart, line chart and electrophoresis gel figure etc. involved in red-light core gene expressions from published articles or websites.

Second step: Convert the electrophoresis gel figures into a grayscale or black and white array. The number of pixels occupied by the black or gray arrays are regarded as relative gene expression. The data of bar or line charts can be directly extracted and transformed into relative expressions. The detailed steps can be found in the supplementary material of the published paper [70].

Third step: Rescale the extracted relative expressions data to a reasonable range.

S5.2 Numerical method

In general, nonlinear differential equations do not have analytical solutions. We usually use a numerical method to obtain a numerical solution of high accuracy. The most successful methods during more than half a century were the 4th order methods of Kutta [71]. Here, we choose the 4th-order Gauss method for model simulation. The mathematical formula and the corresponding Butcher table of the used Gauss method are given below.

Mathematical formula:

$$\begin{cases} Y_1 = y_n + h\sum_{j=1}^{2} a_{1j} f(x_n + c_j h, Y_j) \\ Y_2 = y_n + h\sum_{j=1}^{2} a_{2j} f(x_n + c_j h, Y_j) \\ y_{n+1} = y_n + h\sum_{i=1}^{2} b_i f(x_n + c_j h, Y_i) \end{cases}$$

Butcher table:

$$\begin{array}{c|cc} \frac{1}{2}-\frac{\sqrt{3}}{6} & \frac{1}{4} & \frac{1}{4}-\frac{\sqrt{3}}{6} \\ \frac{1}{2}+\frac{\sqrt{3}}{6} & \frac{1}{4}+\frac{\sqrt{3}}{6} & \frac{1}{4} \end{array}$$

	$\frac{1}{2}$	$\frac{1}{2}$

S6. Computer codes used for numerical simulation

The python codes for the numerical simulation (Figures 2-9) of the model were attached in a package named as code.rar.
